# The metabolic profiles of cancer stem cells

**DOI:** 10.1186/s13287-026-05014-4

**Published:** 2026-04-13

**Authors:** Zuzana Tylichova, Borivoj Vojtesek, Philip J. Coates

**Affiliations:** RECAMO, Masaryk Memorial Cancer Institute, Zluty kopec 7, 656 53 Brno, Czech Republic

**Keywords:** Cancer stem cells, Metabolism, Glycolysis, Mitochondria, Glucose, Proteasome

## Abstract

Cancer stem cells (CSCs) represent a minor but highly adaptable subpopulation within tumors that drives long-term growth, metastasis, and therapy resistance. Their ability to survive and regenerate under metabolic and therapeutic stress relies on a unique integration of energy flexibility, redox balance, and proteostatic programs. While bulk tumor cells typically favor aerobic glycolysis and high protein turnover, CSCs often exhibit elevated mitochondrial activity, fatty acid oxidation, and selective suppression of proteasome function. These metabolic features support quiescence, stress tolerance, and self-renewal. Beyond energy production, metabolic intermediates such as acetyl-CoA, succinate, and lactate serve as epigenetic cofactors, linking nutrient availability to chromatin remodeling and transcriptional plasticity. Reactive oxygen species and antioxidant responses further tune this balance, shaping the transition between glycolytic and oxidative CSC states. These intrinsic programs are continuously influenced by the tumor microenvironment, where hypoxia, cytokine-driven signaling, and metabolic coupling with stromal and immune cells modulate CSC metabolism and reinforce stemness. Despite rapid progress, major conceptual and methodological gaps still limit our understanding of CSC metabolism and this review highlights these unresolved issues and further outline key contextual factors—including tumor-intrinsic, microenvironmental, systemic, and metastatic cues—that shape CSC metabolism and help explain the divergent observations reported across studies. Understanding this network will be essential for designing combinatorial therapies that target CSC metabolism while accounting for their heterogeneity and plasticity.

## Cancer stem cells

**Fig. 1:**
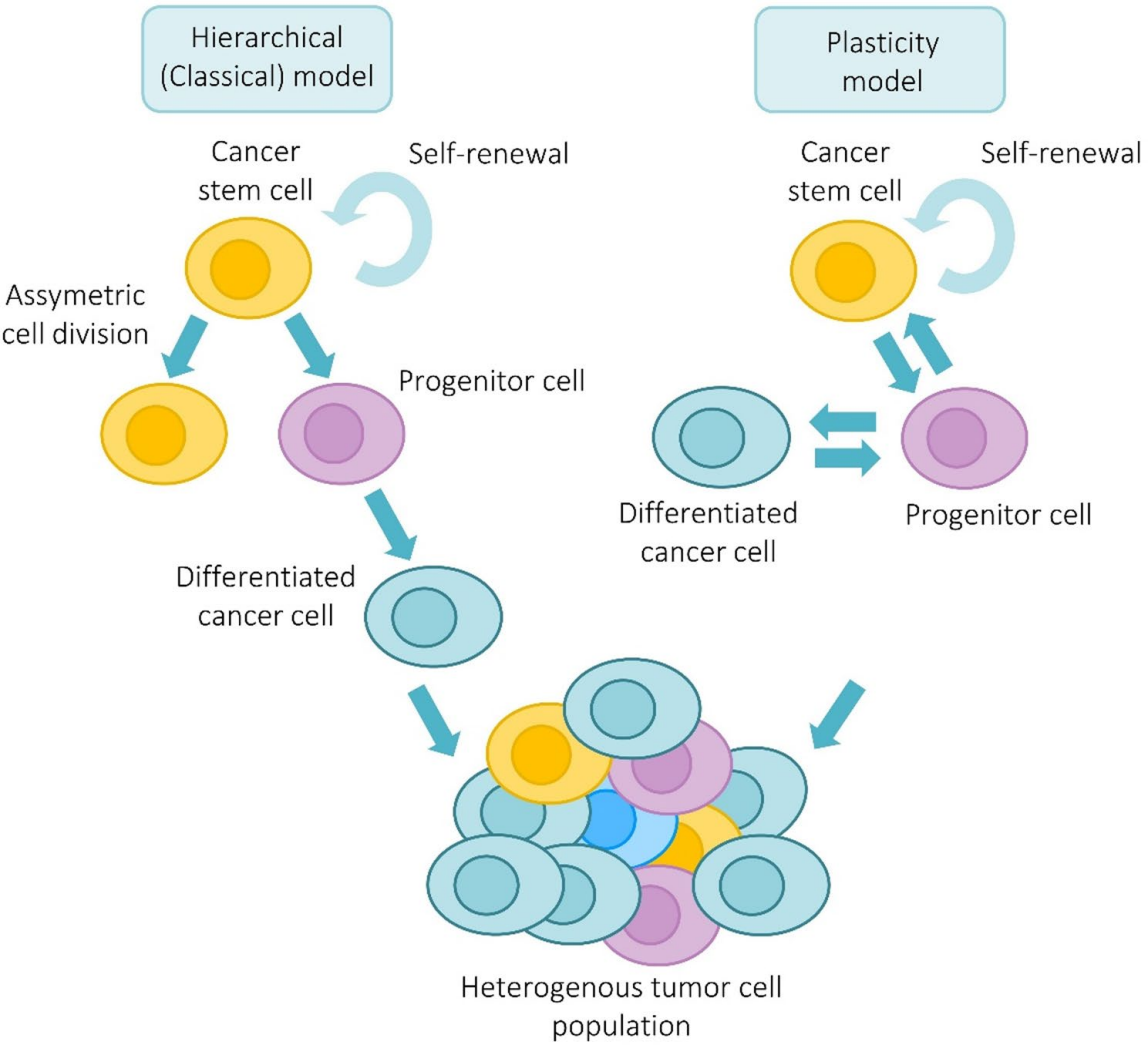
CSC models. Two conceptual models have been proposed to explain the organization and dynamics of CSCs within tumors: the hierarchical (classical) and the plasticity model. In the hierarchical model, only a small subset of cells (CSCs) have the ability to self-renew and drive tumor growth through asymmetric division, generating both CSCs and more differentiated progeny with limited proliferative potential. In contrast, the plasticity model proposes that stemness represents a dynamic state influenced by external signals (e.g., niche, inflammation, tumor microenvironment), cancer cells can transition between CSC and non-CSC states. This may explain tumor adaptability, resistance to therapy, and recurrence. These models are not mutually exclusive; however, increasing evidence suggests that stemness is a dynamic and reversible state shaped by cellular plasticity

Tumors are comprised of a mixture of non-malignant (stromal) cells that form the tumor microenvironment, together with the malignant population of tumor cells. Within the malignant cell population, a subset of cells with stem-like properties is commonly referred to as cancer stem cells (CSCs). These cells are characterized by their capacity for self-renewal and their ability to generate progenitor cells that proliferate rapidly and give rise to the heterogeneous tumor cell populations. Two conceptual models have been proposed to explain the organization of CSCs within tumors: the hierarchical model, in which a defined CSC population drives tumor growth, and the plasticity model, which proposes that stemness represents a dynamic state rather than a strictly defined cellular hierarchy (Fig. 1) [1, 2]. Although early CSC models proposed a rigid hierarchical organization, accumulating evidence has revealed substantial plasticity, whereby non-CSC tumor cells can acquire stem-like properties in response to microenvironmental or intrinsic signals [3–5]. These stemness properties are lost during differentiation and are governed by pathways such as STAT3, NANOG, NOTCH, WNT, and HEDGEHOG, which are highly dysregulated in CSCs due to genetic and epigenetic changes [6]. Similar to normal tissue stem cells, CSCs often represent only a small fraction of the tumor, and are regulated by signals from their microenvironment (the CSC niche) [7, 8]. These cells exhibit relatively slow turnover rate compared to rapidly proliferating progenitor and tumor cells, which contributes to their resistance to therapies targeting fast-growing cells [9]. CSCs exhibit multiple mechanisms of therapy resistance, including efficient DNA damage response systems and elevated expression of drug-efflux transporters that reduce intracellular accumulation of chemotherapeutic agents [10]. Additional CSC-associated traits include altered signaling activity, epithelial–mesenchymal transition (EMT), increased anti-apoptotic and autophagy programs, and epigenetic modifications such as DNA methylation and histone remodeling [11, 12].

## Identifying CSCs—markers and functional assays

CSCs were first identified in leukemia via their CD34^+^/CD38^−^ phenotype in 1994 [13]. The first reports of CSCs in solid tumors were published in 2003, demonstrating that CD44^+^/CD24^−^ breast cancer cells were able to self-renew and re-establish tumors with a range of partially differentiated cell types when transplanted into immunodeficient mice [14]. Subsequently, CSCs from solid tumors have been identified using a variety of assays [15]. These include the presence of specific cell surface proteins, such as Lgr5 [16], CD24/CD44 [17], and CD133 [18], high aldehyde dehydrogenase (ALDH) activity [19], the ability to efflux dyes to produce a side population in flow cytometry [20], the ability to form spheres in suspension culture [21], the production of colonies with holoclone morphology [22, 23], or by forming xenograft tumors in immunodeficient mice [24]. However, none of these is a universal marker of the CSC population in all tumor types, or in different tumors of the same type. For example, CD44^+^/CD24^−^ and ALDH^+^ cells are widely used as CSC markers in breast cancer. However, luminal breast cancers are enriched in CD44^−^/CD24^+^ cells, whereas tumor cells in basal/mesenchymal breast cancers are enriched in the CD44^+^/CD24^−^ phenotype, and the remaining basal/epithelial tumor types are often positive for both markers. ALDH is found mainly in HER2-overexpressing and basal breast cancers [25]. Moreover, markers identify a distinct sub-population of CSCs within a single tumor [11, 26], and ALDH^+^ “epithelial-like” CSCs may transition into “basal-like” CD44^+^ CSCs [27, 28], and vice versa. In addition, CD44/CD24 and ALDH are associated with different tumor characteristics: A high CD44/CD24 ratio is related to cell proliferation and tumorigenesis, while ALDH is an indicator of metastasis [29]. Within these CSC subtypes, breast adenocarcinoma CSCs may express the stem cell marker ΔNp63, which associates with the basal CSC type, while ALDH in these tumors is associated with a lack of ΔNp63 [30] and prostate adenocarcinomas may also show a ΔNp63/basal-like CSC subpopulation [31]. Importantly, ΔNp63 transcriptionally regulates CD44 [32], providing at least one mechanism for inducing specific CSC phenotypes within an individual tumor.

However, the defining feature of CSCs is not marker expression but functional competence—the ability to self-renew and generate heterogeneous progeny. The plastic nature of these cells limits the reliability of static biomarkers, reinforcing the importance of integrated, functionally validated methodologies [33]. Accordingly, serial tumor transplantation remains the functional gold standard for CSC identification, as it directly assesses self-renewal and long-term tumor propagation in vivo. In contrast, most surface and metabolic markers enrich but do not exclusively define CSC populations, reflecting the dynamic and context-dependent nature of CSC phenotypes [34]. Recent technological advances have further challenged the view of CSCs as a fixed cellular population. Lineage-tracing studies have demonstrated that tumor cells can dynamically transition between stem-like and non-stem states in vivo, while single-cell transcriptomic analyses reveal continuous cellular state spectra rather than discrete CSC populations. In addition, organoid and patient-derived tumor models enable functional interrogation of stemness under physiologically relevant conditions [35, 36]. Alongside these modern approaches, classical in vitro assays have also provided insight into cellular hierarchies within epithelial tumors. Cell lines generated from carcinomas consistently produce in vitro colony patterns similar to those produced by the stem and amplifying cells of normal epithelia. From the differing types of colony morphologies, it is possible to predict both the growth potential of their constituent cells and their patterns of macromolecular expression. Colonies termed holoclones, consisting of small, tightly packed cells forming round colonies with high proliferative potential are thought to contain stem cells. Meroclones contain larger and less tightly packed cells than holoclones and have less proliferative potential and less ability to self-renew, corresponding to transit amplifying cells. Paraclones are identified as loose irregularly shaped colonies, consisting of large, flattened cells, have low proliferative potential and correspond to early differentiating cells [37]. Major difference between holoclones and meroclones derived from a cancer cell line could be the proportion of CSCs within each colony [38]. Holoclone morphology is associated with higher levels of stem cell-related molecules such as β-integrin, E-cadherin, β-catenin and CD44 [37, 39]. Patterns of colony morphologies correlate with standard identification methods, such as the presence of dye-excluding side population, the CSC marker CD44, and their ability to grow as spheroids [40]. These observations reflect the inherent heterogeneity and plasticity of CSC populations. By definition, CSCs exhibit the capacity for differentiation, and may transition between cell states, thus contributing to intratumoral heterogeneity.

This heterogeneity often reflects differences in differentiation and developmental programs programs resembling those of the tissue of origin, and is frequently driven by transcriptional heterogeneity rather than genetic mutations [41]. Importantly, intratumoral heterogeneity represents a major challenge for cancer therapy, particularly through transcriptional programs regulating proliferation and differentiation meta-programs [41]. This phenotypic plasticity also includes EMT, which promote invasion and metastasis, and the reverse mesenchymal–epithelial transition (MET), which facilitate colonization at secondary sites [42]. Different CSCs subpopulations exhibit different EMT/MET patterns. For example, in breast cancer, MET CSCs are characterized by high ALDH activity and enhanced proliferative capacity, whereas EMT CSCs identified by CD44^+^/CD24^−^ are slow cycling and quiescent [27]. Metastatic potential is therefore not defined by a single universal marker but rather by functional capacity to invade, disseminate, and colonize distant organs. Although EMT-associated markers such as reduced E-cadherin and increased N-cadherin or Vimentin with upregulation of EMT transcription factors (Snail, Slug, Twist, and Zeb1/2) are commonly linked to invasion, they do not exclusively define metastatic potential. Similarly, circulating tumor cells are frequently detected using epithelial markers such as EpCAM or cytokeratins, which reflect detection strategies rather than true metastatic potential [43]. Increasing evidence indicates that stemness represents a reversible cellular state linked to epithelial–mesenchymal plasticity, where EMT programs promote stem-like traits and contribute to tumor progression, metastasis, and therapy resistance [44, 45]. EMT-associated metabolic changes are discussed in the context of metastatic dissemination in “Metastatic dissemination and organ-specific metabolic adaptation” section. Overall, current CSC markers identify overlapping but distinct subpopulations that reflect the tumor type, cellular context, and methodological approach rather than a single universal CSC identity. CSC phenotypes therefore represent dynamic cellular states, and marker expression may fluctuate depending on cellular plasticity, microenvironmental cues, and culture conditions.

## Overview of metabolic hallmarks of CSCs

**Fig. 2:**
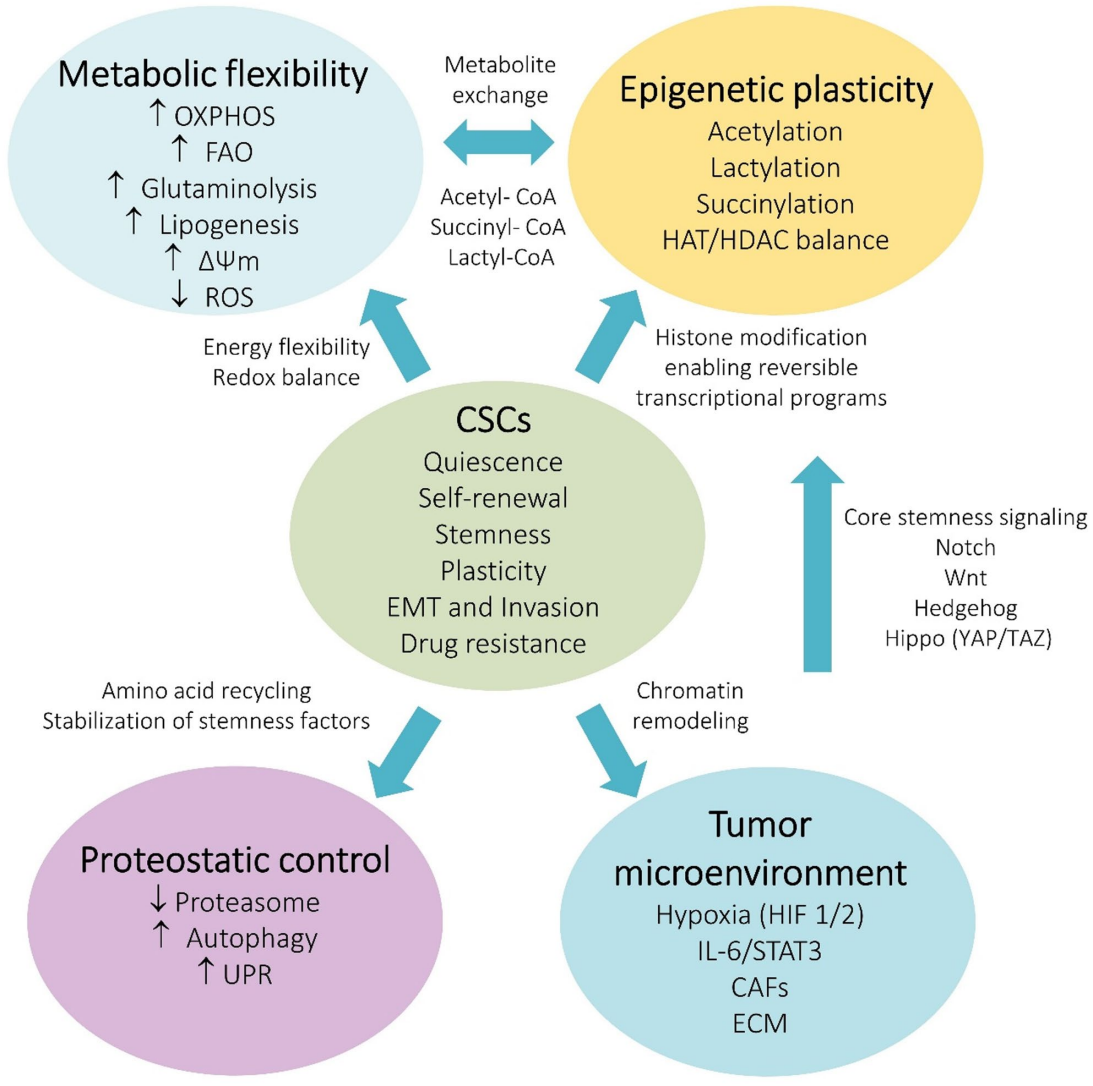
Metabolic reprogramming aspects in CSCs. CSCs integrate metabolic flexibility, epigenetic plasticity and proteostatic control. Tumor microenvironmental signals reinforce these programs and maintain CSC identity, survival and therapy resistance. *CAFs* cancer associated fibroblasts, *ECM* extracellular matrix, *EMT* epithelial-to-mesenchymal transition, *FAO* fatty acid oxidation, *HAT* histone acetyltransferase, *HDAC* histone deacetylase, *ROS* reactive oxygen species, *OXPHOS* oxidative phosphorylation, *UPR* unfolded protein response

Cancer cells acquire metabolic adaptations that enable the utilization of both conventional and alternative nutrient sources to fuel biomass production, sustain uncontrolled proliferation, and modulate cell fate within the tumor microenvironment [46]. Although both cancer cells and CSCs exhibit metabolic flexibility, their priorities diverge—bulk tumor cells primarily optimize pathways for rapid growth, whereas CSCs display a distinct metabolic organization that supports their capacity for long-term survival, self-renewal, stress resilience, tumor re-initiation and therapy resistance [47]. Key metabolic hallmarks of CSCs include enhanced redox balance, efficient mitochondrial function, and the use of metabolic intermediates as signaling or epigenetic regulators. Glucose and glutamine remain major carbon sources, but their fate differs from that in non-CSCs. Lipid metabolism is frequently rewired to sustain membrane synthesis and signaling lipids important for stemness. CSCs maintain a finely tuned ROS homeostasis, sufficient for signaling but low enough to prevent oxidative damage. Metabolite-driven epigenetic modifications create a feedback loop between metabolism and transcriptional programs controlling stemness. CSCs exhibiting high mitochondrial membrane potential, elevated succinate dehydrogenase activity, and low lactate dehydrogenase levels rely predominantly on oxidative phosphorylation, which supports their long-term survival, quiescence, and resistance to stress and therapy through efficient energy production and enhanced metabolic flexibility. Reduced proteasome activity may contribute to the stabilization of stemness-associated transcription factors, maintenance of a quiescent and stress-resistant state, and protection against oxidative and proteotoxic stress, thereby promoting their survival and therapy resistance. These features make CSC metabolism not a single, uniform profile but a dynamic continuum shaped by tumor type, microenvironmental conditions, and therapeutic pressure (Fig. 2). Such metabolic plasticity becomes particularly evident during metastatic dissemination, where tumor cells must adapt to distinct metabolic challenges at each stage of the metastatic cascade. Understanding these metabolic hallmarks provides a framework for interpreting the divergent findings in subsequent sections and for identifying metabolic vulnerabilities that can be targeted therapeutically.

## Energy and nutrient metabolism

### Glucose metabolism in CSCs

**Fig. 3:**
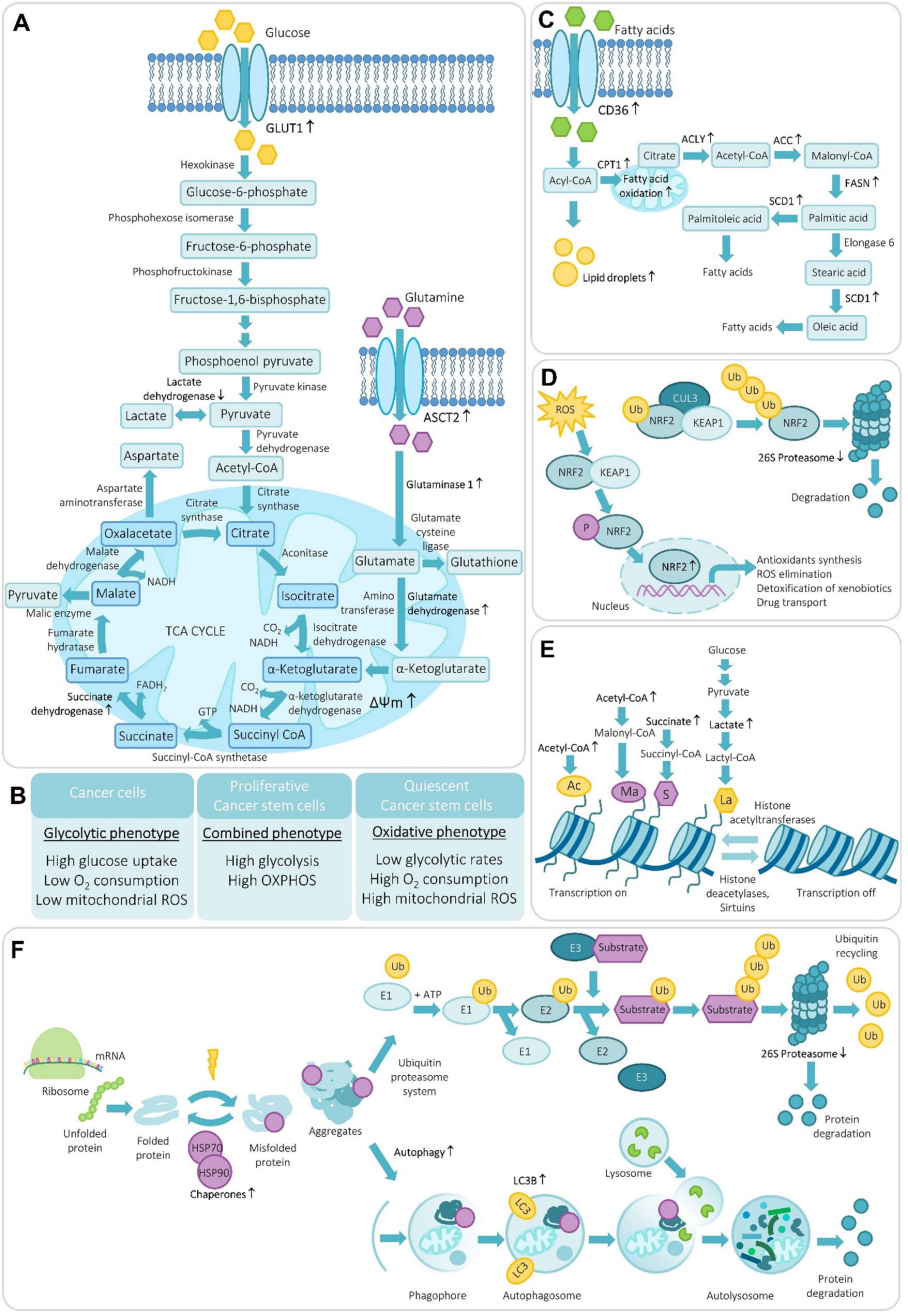
Metabolic features of CSCs. **A** Glucose and glutamine support anabolic metabolism. In CSCs, increased expression of GLUT transporters and glutamine uptake can fuel OXPHOS and biosynthetic pathways. **B** CSCs show pronounced metabolic flexibility, shifting between glycolysis and OXPHOS depending on proliferative or quiescent states. **C** CSCs enhance lipid synthesis and storage via ACLY, ACC, FASN, ACS, and SCD1 enzymes, contributing to membrane remodeling, signaling, and survival. Fatty-acid oxidation, initiated by CPT1, provides ATP and supports survival under stress. **D** CSCs maintain low ROS by activating the Nrf2, which induces transcription of antioxidant and detoxifying enzymes. Controlled redox balance is essential for CSC survival and therapy resistance. **E** Metabolite-derived post-translational modifications (acetylation, lactylation, succinylation, etc.) link metabolic state to epigenetic regulation of stemness. **F** CSCs rely on enhanced proteostasis—reduced proteasome activity, elevated chaperones (e.g., HSP70, HSP90), and selective autophagy—to maintain protein quality and stemness under stress. Arrows indicate CSC-specific upregulation or downregulation of each pathway component. *ACC* acetyl-CoA carboxylase, *ACLY* ATP citrate lyase, *ACS* acyl-CoA synthetase, *ASCT2* Alanine/Serine/Cysteine Transporter 2, *CPT1* carnitine palmitoyltransferase 1, *E1* ubiquitin-activating enzyme, *E2* ubiquitin-conjugating enzyme, *E3* ubiquitin ligase, *FASN* fatty acid synthase, *GLUT* glucose transporter, *HSP* heat shock protein, *NRF2/Keap1* nuclear factor erythroid 2-related factor 2/Kelch-like ECH-associated protein 1, *OXPHOS* oxidative phosphorylation, *ROS* reactive oxygen species, *SCD1* Stearoyl-CoA desaturase-1, *TCA* tricarboxylic acid cycle, *Ub* ubiquitin, *ΔΨm* mitochondrial membrane potential

Glycolysis is an oxygen-independent metabolic pathway that occurs in the cytosol, utilizing 2 and generating 4 molecules of ATP from the conversion of glucose into pyruvate, which in the absence of oxygen is then used to produce lactate by the enzyme lactate dehydrogenase (LDH) (Fig. 3A). In the presence of oxygen, pyruvate is transported into mitochondria and converted into acetyl-CoA that enters into and is metabolized through the tricarboxylic acid (TCA) cycle, acting as the terminus for oxidation and producing carriers that donate electrons onto the electron transport chain in the inner mitochondrial membrane to produce up to 34 ATP molecules, an order of magnitude higher level than is produced from glycolysis. In cancer cells, the glycolytic phenotype might predominate over oxidative phosphorylation (OXPHOS). CSCs appear to be metabolically plastic and use glycolysis, OXPHOS or both, depending on whether they are in a proliferative or quiescent state, and and on their energetic requirements [47–49] (Fig. 3B). While several studies have reported that CSCs exhibit elevated glucose uptake and glycolytic flux compared with non-CSCs, others describe CSCs as relatively glycolysis-independent and preferentially reliant on OXPHOS.

In contrast to the high growth and proliferation rate of most cancer cells, CSCs are considered to be relatively quiescent and, therefore, do not require the same high levels of glucose or glutamine to produce intermediates for anabolism through glycolysis [50]. Thus, relative metabolic activities with reduced reliance on aerobic glycolysis could be a distinguishing characteristic of CSCs compared to the tumor bulk. On the other hand, long-lived cells such as CSCs should avoid oxidative damage associated with aerobic respiration in the mitochondria to ensure their extended survival [51]. The metabolic phenotype of CSCs has been the subject of intense investigation in the past decade, leading to observations that CSCs can rely primarily either on glycolysis or on OXPHOS, mainly depending on the tumor type, with a reliance on mitochondrial function representing a critical aspect of CSCs [47]. Growing evidence shows that CSCs often have a preference for mitochondrial oxidative metabolism, are less glycolytic, consume less glucose, produce less lactate and maintain higher ATP levels than their progeny [52, 53]. Also, TCA cycle metabolites including pyruvate, citric acid, malic acid and succinic acid are upregulated in CSCs, indicating differences in major energy production pathways [54]. Several studies reported reduced glucose consumption in CSCs [55] and restriction of glycolysis has been shown to further enrich the CSC population [56]. Sphere-forming and CD133⁺ CSCs, in particular, rely predominantly on oxidative phosphorylation for their energy production [57, 58]. Leukemic CSCs are also characterized by low levels of ROS and a less glycolytic phenotype [59], and increased OXPHOS is required for stemness maintenance in some tumors [60].

On the other hand, CSCs may use glycolysis as the preferred metabolic process. Glycolysis-related enzymes, including hexokinase II and glucose-6-phosphate isomerase, as well as glucose transporter 1 (GLUT1), are elevated in some CSCs [20], and GLUT1 inhibition reduces the self-renewal and tumor-initiating capacity of glioma, pancreatic and ovarian cancer cells [61]. The CSC marker CD133 is a regulator of glucose transport, and silencing CD133 inhibits GLUT1-mediated glucose transport and tumor invasiveness [62]. Similarly, the stem cell marker Nanog decreases OXPHOS and increases glycolysis and fatty acid oxidation to support both self-renewal and drug resistance [63], and the stem-cell transcription factor p63 controls cancer cell metabolism and reprogramming through direct transcriptional activation/repression of target metabolic genes [64, 65]. Other studies have shown increased glucose uptake and lactate production, together with a decrease in mitochondrial respiration in CSCs compared with their non-CSC counterparts [66, 67] and a switch from OXPHOS to aerobic glycolysis was reported to be essential for the CD44^+^CD24^low^EPCAM^+^ phenotype of breast CSCs due to decreased ROS levels [68]. EMT CSCs seem to favor glycolysis, showing reduced oxygen consumption, decreased mitochondrial mass and membrane potential, lower ROS production and higher antioxidant capacity compared with the epithelial-like subtype of CSCs [69–71]. Conversely, ALDH^+^ populations of CSCs express more glycolytic enzymes than the CD44^high^/CD24^low^ CSC population [72]. Our data demonstrate that high glucose uptake correlates with high mitochondrial membrane potential; however, colonies derived from cells with high mitochondrial membrane potential exhibit heterogeneous metabolic parameters [73].

Over the last decades new biochemical technologies have provided powerful tools [74], but the lack of appropriate and consistent markers has limited the characterization of the metabolic activities of CSCs [75]. Thus, the link between glycolysis and mitochondrial respiration in CSCs is not straightforward. Apparent differences across studies likely originate from the different CSC subtypes observed within tumors, different proliferative states of quiescent versus activated CSCs, different CSC isolation methods/markers, and/or the organotropic metastatic capacities of CSCs [47, 76–79]. Reported differences in glucose uptake may also reflect experimental context and assay choice, which can invert the metabolic phenotype. For example, increasing cell density in prostate cancer cells induces a switch from glycolysis to OXPHOS and enhances CSC-like traits, while inhibition of the Warburg effect with dichloroacetate prevents this shift [80]. Fluorescent glucose analogs such as 2-NBDG are strongly influenced by extracellular glucose concentration and transporter competition, so uptake rates vary with medium composition and require careful standardization and calibration [81]. Moreover, hypoxia increases 18 F-FDG uptake in cancer cells, meaning that oxygen is a major determinant of measured glucose utilization [82]. Model geometry also matters: 3D spheroids exhibit markedly different glucose and energy fluxes compared with 2D monolayers, producing divergent readouts even for the same cell line [83, 84]. Finally, carbon source selection can reprogram metabolism: replacing glucose with galactose forces reliance on OXPHOS and alters apparent glucose dependency [85]. These variables can explain why some studies observe high glucose uptake/glycolysis in CSCs, whereas others report OXPHOS-dominant phenotypes, underscoring the need to report and control assay conditions when comparing glucose metabolism across models. Overall, CSCs can adopt either glycolytic or OXPHOS-dominant programs, and the balance between these states appears to be highly context-dependent, reflecting cell state, microenvironment, and methodological differences across studies.

### Glutamine and amino-acid metabolism in CSCs

Glutamine is a nonessential amino acid that participates in the TCA cycle and the biosynthesis of nucleotides, glutathione, and other nonessential amino acids (Fig. 3A). Cancer cells have an unusually high rate of glutamine consumption, and glutamine deprivation suppresses cancer growth and induces cell death [86, 87]. Indeed, standard cell culture conditions include the addition of glutamine to the medium to ensure an adequate supply. Glutamine enters cells through the amino acid transporter SLC1A5 (also known as ASCT2), and is converted to glutamate in the mitochondria through a deamination reaction catalyzed by glutaminase. Glutamate is converted to the TCA cycle intermediate α-ketoglutarate by either glutamate dehydrogenase or by alanine or aspartate transaminases [88]. CSCs may show increased levels of glutaminase and glutamate-oxaloacetate transaminases, and glutamine deprivation decreases stemness [89, 90]. Glutamine transporter SLC1A5 drives malignant behavior in glioma cells by increasing glutamine uptake and fueling downstream metabolic and signaling pathways linked to proliferation, survival, and stem cell-like properties [91]. Glutamine supports the CSC phenotype by promoting glutathione synthesis to maintain redox balance, and glutamine deprivation increases intracellular ROS levels and reduces the CSC population [92]. Glutamine catabolism may also contribute to CSC maintenance through the 2-oxoglutarate dependent chromatin-modifying dioxygenase [93]. In primary tumors, upregulation of glutaminase is associated with advanced clinicopathological features and a stemness phenotype, and targeting glutaminase attenuates tumorigenicity and stemness properties, implying that glutaminase could serve as a therapeutic target to eliminate CSCs [94–96].

As with glucose metabolism, opposite effects of glutamine have also been seen in CSCs, where glutamine restriction promotes stemness and carcinogenesis by decreasing intracellular α-ketoglutarate levels, whereas α-ketoglutarate supplementation promotes cellular differentiation through epigenetic modifications to DNA and histone methylation [97]. In addition, glutamine deprivation has been associated with increased recurrence and metastasis [98]. These two studies relate specifically to colorectal cancer, which may suggest a tumor-type specificity for glutamine in cancer. Thus, the role of glutamine in CSCs is complex, and distinct glutamine downstream products, pathways and enzymes have been implicated in different CSC subpopulations [99]. Glutamine metabolism is a context-dependent regulator of CSC phenotypes. In many tumor models, CSCs rely on SLC1A5-mediated glutamine uptake and glutaminase-driven glutaminolysis to feed the TCA cycle [100], however, several CSC subsets use alternative carbon sources or activate compensatory pathways [101].

In addition to glutamine, multiple amino acids have emerged as important regulators of CSC metabolism, including serine and one-carbon metabolism, branched-chain amino acid (BCAA) catabolism, and context-specific utilization of lysine, threonine, and methionine [102–105]. These pathways collectively link amino acid availability to redox balance, translational control, and chromatin remodeling. *De novo* serine biosynthesis sustains tumor-initiating capacity in non-small-cell lung carcinoma by supporting nucleotide production, redox homeostasis, and chemoresistance [106]. Beyond its metabolic role, serine availability can influence stem cell fate through α-ketoglutarate-dependent epigenetic regulation [107]. Consistently, phosphoglycerate dehydrogenase (PHGDH), a key enzyme of the serine synthesis pathway, enables stem-like gastric cancer cells to survive glutamine deprivation by activating a serine-driven mitochondrial folate pathway that generates NADPH and maintains redox balance [90]. Elevated PHGDH expression in glioblastoma stem cells has been associated with enhanced self-renewal, activation of one-carbon metabolism, and aggressive tumor progression [102]. Threonine and lysine further exemplify the integration of amino acid metabolism with epigenetic and translational regulation. In glioblastoma stem cells, threonine serves as a substrate for t⁶A tRNA modification, promoting translational reprogramming and tumor growth [108]. Similarly, rewired lysine uptake and catabolism in glioblastoma stem cells increase intracellular crotonyl-CoA levels, leading to enhanced histone lysine crotonylation. This epigenetic remodeling supports CSCs maintenance and tumor growth [105]. Methionine availability also critically shapes CSC function. Methionine restriction sensitizes CD133⁺ colorectal CSCs to chemotherapy through suppression of c-Myc expression, highlighting its role in regulating stemness and drug resistance [109]. In clear cell renal carcinoma, tumor-associated pericytes supply methionine to CSCs, providing supportive niche for CSCs [110]. BCAA metabolism represents another axis linking amino acid utilization to CSC maintenance. The branched-chain aminotransferases (BCAT1/2) are frequently upregulated in cancer [111], and METTL16-mediated m^6^A modification promotes leukemia stem cell self-renewal by enhancing BCAT expression and reprogramming BCAA metabolism in acute myeloid leukemia [104]. In parallel, BCAT1 contributes to leukemia stem cell maintenance through α-ketoglutarate depletion and consequent alterations in DNA methylation [112]. Collectively, these findings indicate that amino acid dependencies in CSCs extend far beyond glutamine and reflect a coordinated integration of metabolic flux, redox regulation, translational control, and epigenetic remodeling that supports cellular plasticity and therapeutic resistance.

### Lipid metabolism in CSCs

Cancer cells fulfil their demands for lipids by either increasing exogenous lipid uptake or by endogenous production through *de novo* synthesis. A series of lipid synthesis enzymes are upregulated in CSCs, including ATP citrate lyase (ACLY), acetyl-CoA carboxylase (ACC), fatty acid synthase (FASN), acyl-CoA synthetase (ACS) and stearoyl-CoA desaturase 1 (SCD1) [113] (Fig. 3C). Fatty acid biosynthesis is mainly regulated by sterol regulatory element-binding proteins (SREBPs) and peroxisome proliferator-activated receptors (PPARs) [114]. PPARs are ligand-activated transcription factors belonging to the nuclear hormone receptor superfamily, comprising three subtypes—PPARα, PPARγ, and PPARβ/δ—each exhibiting tissue-specific expression [115]. The PPAR seems to be critical for CSC properties, as PPARγ inhibition reduces the ALDH^+^ population of breast CSCs [116], and PPAR*γ* agonists reduces the expansion of CD133^+^ glioma CSCs [117]. Treatment of hematopoietic CSCs with PPARδ agonists improves stem cell functions in promyelocytic leukemia through fatty acid oxidation [118]. PPARδ is linked to the pluripotency factor Nanog, which represses OXPHOS genes and ROS production while promoting fatty acid oxidation to sustain liver CSC self-renewal and drug resistance. In turn, restoring OXPHOS activity and inhibiting fatty acid oxidation re-sensitize CSCs to chemotherapy. Through its interaction with Nanog, PPARδ further enhances fatty acid oxidation and supports the maintenance of this resistant metabolic state [63]. PPAR signaling contributes to the broader regulation of lipid metabolism, including lipid storage and membrane remodeling. Emerging lipidomic analyses indicate that phospholipid metabolism also contributes to CSC biology. In pancreatic CSCs, lipidomic profiling revealed increased fatty acid elongation and accumulation of long-chain unsaturated fatty acids together with remodeling of mitochondrial cardiolipin species, linking lipid metabolism to stemness-associated metabolic programs [119]. Similarly, in acute myeloid leukemia, cardiolipin remodeling and phosphatidylserine balance regulate leukemia stem cell maintenance and represent potential metabolic vulnerabilities [120]. Remodeling of membrane phospholipids further contributes to the regulation of membrane fluidity and signaling in CSCs [121]. For example, altered phospholipid remodeling regulates intestinal stem cell proliferation through cholesterol biosynthesis, where increased membrane saturation stimulates cholesterol production and promotes stem cell expansion and tumorigenesis [122]. To sustain membrane remodeling and biogenesis, CSCs frequently enhance *de novo* fatty acid synthesis, with elevated FASN expression supporting membrane production. Pharmacological inhibition of FASN reduces stemness marker expression and limits CSC proliferation and migration [123]. Lipid droplets serve as dynamic reservoirs of neutral lipids that supply lipids for membrane synthesis and signaling. Increased lipid droplet content has been observed in several solid tumor CSCs, including CD133^+^ colorectal [124], ALDH^+^/CD133^+^ ovarian [125], CD44^+^/CD24^−^ breast [126] and CD133^+^CD44^high^ pancreatic CSCs [127]. Colorectal CSCs with more lipid droplets showed a higher tumorigenic capacity upon xenotransplantation [124]. Fatty acids are strictly regulated by CSCs to maintain their self-renewal ability and therapy resistance [128]. Ovarian ALDH^+^ CD133^+^ CSCs contain unusually high levels of unsaturated lipids, a pattern also seen in CSC-enriched spheroids. This is linked to elevated SCD1, the predominant desaturase that converts saturated fatty acids into monounsaturated fatty acids. In sphere-forming CSC cultures, levels of all measured fatty acids—including palmitic, palmitoleic, stearic, oleic, linoleic, arachidonic, and docosahexaenoic acids—were elevated compared to adherent cells. Pharmacological inhibition of SCD1 abolished CSC populations, impaired sphere formation, and suppressed tumor initiation in vivo. Furthermore, nuclear factor κB (NF-κB) directly regulates lipid desaturases, and inhibition of these enzymes was shown to inactivate NF-κB signaling [125]. Inhibition of SCD1, NF-κB, and ALDH1A1 in CSCs reduces unsaturated lipid levels and impairs spheroid formation, resulting in reduced stemness [54]. In hepatocellular carcinoma spheres, cooperation between SCD1 and PPARα reinforces CSC properties by promoting nuclear accumulation of β-catenin, whereas SCD1 inhibition disrupts sphere formation, decreases CSC marker expression and prevents β-catenin translocation [129]. Interestingly, this pattern is not universal—in human breast CSCs, mass spectrometry revealed lower palmitoleic acid levels compared with non-CSCs, consistent with reduced SCD1 expression [130]. In gastric cancer, SCD1 further regulates CSC properties and drives metastasis through the activation of the Hippo/YAP pathway [131]. Analysis of The Cancer Genome Atlas data shows positive correlations between ovarian CSC markers (CD44^+^, CD133^+^, ALDH1A1^+^) and lipid metabolism enzymes, including CD36, ACC, SCD1, and carnitine palmitoyltransferase (CPT1) [132, 133]. Altogether, lipid and fatty acid metabolism emerges as a critical determinant of CSC maintenance and plasticity, providing both structural components and signaling cues that sustain their stem-like and adaptive phenotype.

## Mitochondrial and enzymatic regulation

### Mitochondria

Mitochondrial membrane potential (MMP), which reflects mitochondrial proton pump activity during OXPHOS, is thought to correlate with cell differentiation status, tumorigenicity and malignancy [134–138]. CSC mitochondria are frequently characterized by increased mass and membrane potential, elevated oxygen consumption, and higher ROS production compared with the bulk tumor cells, which primarily rely on glycolysis for energy generation [52, 58]. However, several studies describe CSCs with higher MMP but low ROS levels, perinuclear mitochondrial localization, reduced mtDNA content, decreased oxygen and glucose consumption, and lower intracellular ATP concentrations—features consistent with a distinct, OXPHOS-dependent metabolic state [55, 134, 139]. Moreover, the CSC marker CD133 has been associated with cells exhibiting higher MMP [55], and both primary human breast CSCs and CSCs derived from breast cancer cell lines display increased mitochondrial mass [58, 140–142]. However, as with many aspects of cell metabolism discussed here, the relationship between MMP and stemness is context dependent. Normal hematopoietic stem cells maintain low MMP [135], but MMP shifts markedly upon differentiation [134]. Given this mitochondrial plasticity, it becomes essential to consider the TCA cycle enzymes, particularly dehydrogenases, that orchestrate the redox and metabolic cues feeding into CSC maintenance.

### Dehydrogenases and TCA regulation

CSCs have also been linked to altered activity of metabolic enzymes involved in glucose metabolism. Dehydrogenases are oxidoreductases that catalyze substrate oxidation by transferring hydrogen to electron acceptors such as NAD⁺/NADP⁺ or flavin cofactors (FAD/FMN) [143]. A key glycolytic enzyme, lactate dehydrogenase (LDH), converts pyruvate to lactate using NADH as a cofactor and serves as a marker of fermentative/aerobic glycolysis. Consistent with the well-established reliance of many tumors on aerobic glycolysis, LDH is upregulated across numerous human malignancies [144], maintains stemness and metastatic potential and its silencing disrupts EMT-related transitions, reduces ALDH⁺ and CD44⁺/CD24⁻ stem-like populations [145]. LDH inhibition suppresses sphere formation and promotes differentiation and death of glioblastoma stem cells [146]. Consistent with this, glioblastoma CSCs sustain stemness under glycolytic blockade by activating compensatory senescence and autophagy pathways, whereas combined inhibition of glycolysis and autophagy disrupts this compensatory loop and induces apoptotic cell death [147]. Beyond glioblastoma, LDH also drives tumor progression by enhancing EMT and upregulating stemness-associated genes in bladder cancer cells [148].

Succinate dehydrogenase (SDH) catalyzes the oxidation of succinate to fumarate with the reduction of ubiquinone to ubiquinol in the inner mitochondrial membrane [149], and is involved in both the TCA cycle and the electron transport chain, and can therefore be used as an indicator of cellular OXPHOS activity. Although direct evidence linking SDH to CSC maintenance is still limited, several studies implicate SDH dysfunction in promoting stemness-associated metabolic programs. Loss or reduced expression of SDH shifts cancer cells toward aerobic glycolysis, increases succinate accumulation, and enhances malignant behavior, features often associated with CSC-like states [150]. Functional SDH deficiency has been shown to rewire mitochondrial metabolism and increase cellular adaptability under metabolic stress, thereby creating conditions for stemness and survival of therapy-resistant subpopulations [151]. Moreover, SDH inhibition or succinate accumulation drives EMT and stemness through epigenetic reprogramming, including hypermethylation, linking SDH dysfunction directly to enhanced cancer cell plasticity [152, 153].

Isocitrate dehydrogenase (IDH) catalyzes the oxidative decarboxylation of isocitrate to α-ketoglutarate. In humans IDH exists in three isoforms: IDH1 and IDH2 catalyze the conversion of isocitrate to α-ketoglutarate with concomitant reduction of NADP^+^ to NADPH in the cytoplasm and mitochondria, respectively. IDH3 catalyzes the third step of the TCA cycle while converting NAD^+^ to NADH in the mitochondria [154, 155]. IDH1 and IDH2 are often mutated in cancer, including glioma [156], cholangiocarcinoma [157], chondrosarcoma [158], and acute myeloid leukemia [159], and 12% of glioblastoma patients have a mutation in the *IDH1* gene [160]. Mutations in IDH1 and IDH2 have been shown to promote stemness in leukemia by aberrant conversion of α-ketoglutarate to the analog 2-hydroxyglutarate, which induces a block in differentiation and thereby contributes to stemness and oncogenesis [161]. 2-hydroxyglutarate accumulation impairs DNA methylation by inhibiting 2-oxoglutarate-dependent dioxygenases that are involved in histone demethylation and epigenetic DNA modifications [162]. These mutations also impair hematopoietic and adipocyte differentiation [163]. Positive correlations have been found between the mRNA levels of ovarian CSC markers and glycolytic markers. Datasets from The Cancer Genome Atlas revealed that NOTCH1, CD133, CD44, CD24, and ALDH1A1 positively correlated with enzymes of the TCA cycle. Importantly, the degree of correlation varied between individual markers. It has also been found that aconitase 1, IDH1, IDH3A, succinate-CoA ligase GDP-forming subunit beta and malate dehydrogenase 2 are significantly upregulated in ovarian CSCs [132]. Given that dehydrogenases shape the NAD⁺/NADH and FAD/FADH₂ pools, their dysregulation directly affects cellular redox balance, naturally linking metabolic reprogramming to mitochondrial ROS production in CSCs.

### Reactive oxygen species

ROS, i.e. oxygen species more reactive than free oxygen (O_2_), comprise superoxide (O^·^−), hydrogen peroxide (H_2_O_2_), hydroxyl free radical (HO·) and singlet oxygen. ROS are mainly generated by membrane-bound NADPH oxidases and mitochondrial electron transport chain complexes I–III [164]. Chemotherapy, radioactivity, drugs, toxins, and even smoking can increase ROS levels in the cell [165–167]. At physiological levels, ROS act as signaling molecules required for cellular proliferation, differentiation, and survival, whereas excessive ROS leads to oxidative stress, cellular senescence, apoptosis or carcinogenesis [168], unless it is neutralized by antioxidant enzymes like superoxide dismutase, catalase, glutathione peroxidase, vitamin E, vitamin C, and glutathione [169, 170]. Compared with bulk tumor cells, CSCs contain lower ROS levels and enhanced antioxidant defenses, contributing to their radioresistance. CSCs avoid therapy-induced oxidative stress by enhancing antioxidant and detoxification systems, including free-radical scavengers that neutralize ROS through inhibiting lipid peroxidation, trapping radical species, and boosting intracellular glutathione synthesis [170]. Ionizing radiation kills tumor cells largely through ROS-mediated and DNA damage-induced double strand breaks [166, 171]. Importantly, oxidative stress can drive CSCs out of the stem cell state—high intracellular ROS levels promote loss of stemness and induce differentiation, as shown in human embryonic stem cells [172]. Maintaining ROS at a low level seems to be critical for stem cell function. A growing body of evidence indicates that redox regulation is not only a metabolic hallmark of CSCs but also a central determinant of therapy-induced dormancy. Dormant, treatment-resistant cancer cells rely on tightly controlled antioxidant programs and redox-sensitive signaling to maintain survival, and that disrupting this balance can re-sensitize them to therapy [173]. ROS defense system operates via the adhesion molecule CD44, which is highly expressed in CSCs and serves as commonly used stem cell marker. CD44v, a variant isoform of CD44, increases antioxidative capacity by promoting the synthesis of intracellular glutathione, which functions as an antioxidant, reducing intracellular ROS levels [174]. Some CSCs exhibit low vulnerability to ROS and increased survival rates in response to ionizing radiation compared to non-stem cells due to a more efficient DNA damage response, as has been reported as a reason for the radioresistance of CD133^+^ prostate CSCs [175], and CD133^+^ glioblastoma CSCs [176]. On the contrary, an elevated level of mitochondrial ROS due to fatty acid β-oxidation promoted cancer metastasis by inducing EMT in Lgr5^+^ colon sphere CSCs [177].

Antioxidant and detoxifying genes are regulated by transcription factor Nrf2 and its negative regulator Keap1 [175] (Fig. 3D). Nrf2 is activated in response to ROS, which is linked to high activity and poor clinical outcomes in various types of cancers [178], and Nrf2 silencing sensitizes CSCs to DNA targeting drugs [179]. Nrf2 and its targets, including glutamate-cysteine ligase, aldo-keto reductase, glutathione S-transferase, glutathione peroxidase and NADPH quinone oxidoreductase 1 have been implicated in drug resistance of several cancers by protecting cells from oxidative stress [180, 181]. Interestingly, stem cell markers often interact with Nrf2. Nestin was found to bind to Keap1 in lung cancer, leading to Nrf2 stabilization and increased antioxidants [182]. p63 has also been shown to regulate Nrf2 [183], which in turn modulated glucose and glutamine metabolism to support proliferation and enhance cytoprotection [184]. CD44 overexpression facilitated Nrf2 activation and contributed to the aggressive phenotype and drug resistance of CD44^high^ breast CSCs [185]. CD133^high^ colon cancer spheres displayed Nrf2 activation and enhanced CSC-like properties, and Nrf2 silencing reduced sphere forming capacity [186]. Also ALDH contributes to maintaining low intracellular ROS levels by detoxifying reactive aldehydes, and elevated ALDH activity in CSCs is linked to increased radioresistance [187]. Recent evidence shows that the Keap1-Nrf2 pathway regulates ALDH expression and Nrf2 knockdown suppresses the ALDH⁺ population, diminishes radioresistance, and reduces tumorigenic potential [188], while inhibition of ALDH activity in breast CSCs decreases their resistance to chemo- and radiotherapy [189]. Silencing Nrf2 in ALDH^high^ cells further inhibits CSC-like traits, including stemness markers, chemoresistance, sphere formation, and tumor growth. Conversely, ALDH suppression attenuates Nrf2 activation, thereby diminishing CSC-associated properties specifically in ALDH^high^ cells but not in ALDH^low^ cells [190]. Moreover, preclinical studies suggest that glucose restriction through a ketogenic diet may benefit glioblastoma patients by inhibiting glioblastoma CSC proliferation, inducing apoptosis, and reducing stemness via increased ROS production [191]. By altering mitochondrial metabolism and the ratios of redox-sensitive cofactors such as α-ketoglutarate and NAD⁺, ROS levels can directly influence the activity of epigenetic enzymes, thereby coupling oxidative stress responses to metabolite-dependent chromatin regulation.

## Metabolite-driven epigenetic regulation

Epigenetics is a reversible and dynamic process that regulates gene expression without altering the DNA sequence. Multiples studies have focused on the epigenetic mechanisms responsible for the acquisition of stemness features and CSC evolution [192]. Epigenetic modifications such as DNA methylation, histone modifications, miRNAs or chromatin remodeling may be involved in CSC reprogramming and thus provide survival advantages in the CSC subpopulation, and contribute to tumor initiation, progression, metastasis and response to chemotherapy. Metabolic changes and intermediate metabolites can drive tumor progression by inducing chemical post-translational modifications (Fig. 3E), particularly on histones, which are key epigenetic regulators of diverse biological processes [193, 194]. Metabolic enzymes, especially those that supply acetyl-CoA, also play a key role in sustaining histone acetylation and thereby influencing gene expression and cellular behavior [195]. Acylation of lysine residues has been recognized as an important type of reversible post-translational modifications that impact various regulatory functions such as gene transcription, metabolism, protein homeostasis and signal transduction. About 20 different lysine acylations have been discovered, including acetylation [196, 197], propionylation [198], butyrylation [199], crotonylation [200], succinylation [201], malonylation [202], and glutarylation [203]. Short-chain acyl-CoAs, malonyl-CoA, succinyl-CoA, and glutaryl-CoA are the substrates for the corresponding lysine acylation reactions. These CoAs can be synthesized by their corresponding short-chain acyl salts, malonate, succinate, and glutarate, catalyzed by acyl-CoA synthetases such as succinyl-CoA synthetase and malonyl-CoA synthetase. Additionally, these CoAs can be generated and consumed in the TCA cycle, as well as by metabolism of amino acids and lipids [204].

These modifications are enzymatically regulated by the dynamic balance between “writers,” the enzymes that catalyze the covalent modification of specific residues (histone acetyltransferases) and “erasers,” the enzymes that remove the modification (histone deacetylases, Sirtuins). “Readers” are the effector proteins that bind histones in a modification-specific manner [205]. Recently, lactate has also been found to induce a post-translational modification. Lactate accumulation resulting from the Warburg effect leads to lactylation and may offer insight into mechanism by which lactate regulates non-metabolic cellular processes [196, 197, 206]. Elevated levels of lactylation promote stemness in liver CSCs [207], enhance chemoresistance in colorectal CSCs [208] and induce dedifferentiation into a proliferative CSC state [209]. Likewise, succinylation has recently been shown to promote stemness maintenance in ovarian CSCs [133].

Interaction between epigenetics and metabolism was also observed on the level of methylation [194, 210–212]. Methylation of DNA and histones is mediated by DNA methyltransferases, histone methyltransferases and the cofactor S-adenosylmethionine, which is generated by the methionine cycle. DNA methylation is an epigenetic modification of the 5-carbon cysteine residue in cytosine–guanine (CpG) dinucleotides that act to repress gene expression and is a common mechanism to inactivate tumor suppressor genes in cancer [213]. The interplay between metabolite availability and DNA methylation may result in aberrant DNA methylation that targets genes involved in stem programs in cancer. TCA cycle metabolites—α-ketoglutarate, succinate, fumarate and 2-hydroxyglutarate—facilitate changes to DNA methylation by regulating α-ketoglutarate-dependent dioxygenases to remove the methyl group from DNA [214]. The TET family of dioxygenase enzymes regulate active gene expression via establishment of 5-hydroxymethylcytosine epigenetic marks and are deeply embedded in the pathobiology of acute myeloid leukemia. Leukemia cells have reprogrammed metabolism, and TET3 has been shown to be a key upstream regulator as its overexpression promotes DNA demethylation, induces upregulation of glycolysis-associated genes, and promotes leukemia CSCs [215]. Aberrant DNA methylation in tumors is dynamic and contributes to the transition between active and repressive states of gene transcription [192]. Leukemia stem cells rely on DNA methylation by DNMT1 to silence tumor suppressor genes and maintain their self-renewal and tumorigenic potential, and the abrogation of DNA methyltransferase expression blocked leukemia development [216]. The relevance of DNA methylation in CSC regulation and tumor growth is also illustrated by differences in the methylation status of the *CD133* gene promoter in CD133^−^ and CD133^+^ subpopulations isolated from brain [217] and ovarian cancer [218]. Similarly, p63 is another stem-cell factor that is subjected to differential methylation in the *TP63* gene to control expression of p63 isoforms in cancer [219] .

Analogous to DNA and proteins, RNA can undergo more than 170 post-transcriptional modifications [220]. N^6^-methyladenosine (m^6^A) methylation is the most prevalent, but the consequences remain elusive. Studies also indicate that m^6^A regulators are themselves modulated by epigenetic and/or post-translational modifications, including ubiquitination, SUMOylation, acetylation, methylation, phosphorylation, O-GlcNAcylation, ISGylation, and lactylation, or via noncoding RNAs [221]. Recent studies identified the m^6^A RNA modifying enzymes METTL3 and METTL14 as critical regulators of differentiation in both normal hematopoiesis and acute myeloid leukemia, which expands the roles of the epitranscriptome in maintaining the undifferentiated state in somatic stem cells and human cancer [222]. Consistently, METTL16-mediated m6A modification promotes leukemia stem cell self-renewal by reprogramming BCAA metabolism in acute myeloid leukemia [104]. m^6^A methylation, mediated by the enzyme METTL3, reduces the stability of mRNAs that promote naïve pluripotency. This modification is essential for proper exit from the naïve state and for normal differentiation, where its absence leads to impaired lineage priming and early embryonic lethality [223]. m^6^A also maintains hematopoietic stem cell symmetric commitment and identity [224] and promotes lung adenocarcinoma progression through enhancing glycolysis [225]. Many of these modified residues play important roles in RNA metabolism, including RNA structure, stability and dynamics, splicing, polyadenylation, transport, localization, and translatability. Taken together, metabolites such as acetyl-CoA, lactate and succinate act as epigenetic cofactors, creating a direct biochemical link between cellular metabolism and chromatin states that sustain CSC plasticity.

## Protein homeostasis

Due to their rapid proliferation, cancer cells require high rates of protein synthesis and correspondingly elevated levels of molecular chaperones such as HSP70 and HSP90 to maintain protein folding and survival. Transcriptional activation of heat shock proteins (HSPs) through their master regulator HSF1 is essential for tumorigenesis, as it coordinates protein translation and sustains the anabolic state of proliferating cancer cells [226, 227]. Consequently, HSP inhibitors show therapeutic activity across a wide range of human cancers [228]. In contrast, stem cells, including CSCs, maintain a different proteostatic balance and operate at lower rates of protein synthesis (Fig. 3F). Hematopoietic stem cells synthesize markedly fewer proteins than progenitors or differentiated cells, even if forced to proliferate, and either increasing or decreasing protein synthesis rates impairs the stem cell phenotype, indicating that protein synthesis rate is a defining and necessary feature of stem cells in this system [229]. A similar pattern is observed in epidermal stem cells [230], demonstrating that normal stem cells and CSCs harbor lower protein synthesis rates than committed cells [231]. Dividing CSCs synthesize fewer proteins than their progeny, reduced protein synthesis increases the CSC fraction, and these low-translation CSCs show enhanced sensitivity to specific anti-cancer agents [230, 232]. This reduction in protein output is coupled to an equally restrictive approach to protein turnover—CSCs exhibit lower levels of proteasome-mediated protein degradation, maintaining only the minimum proteostasis required to preserve stemness. CSCs can even be purified by utilizing their low proteasome activity. In these assays, introduction of a fluorescent protein tagged with a proteasome-targeted sequence allows the identification of cells with high fluorescence, representing cells with low proteasome activity [233]. Results have shown that low proteasome activity cells show many CSC-related characteristics, including radio- and chemo-resistance, lower ROS production and increased sphere formation [234]. Importantly, targeted elimination of these cells is sufficient to cause in vivo tumor regression of cancer cell xenografts [235], suggesting therapeutic manipulation of proteasome-low cells as a clinical approach for cancer patients. Proteasome-low cancer cells have also been demonstrated to be slow cycling and responsible for metastasis initiation [236]. These considerations of differences in protein degradation rates in CSCs and cells that make up the tumor bulk may underlie the observations that low proteasome activity characterize CSCs [233, 234], whereas tumor cells are generally addicted to abnormally high proteasome activities [237]. Proteasomes regulate the stem-like property of CSCs via degrading key factors in stemness signaling pathways. Low proteasome activity maintained the stability of repressor element 1-silencing transcription factor (REST) to keep glioblastoma CSCs in a dedifferentiated state [238] and decreases the degradation of Notch intracellular domain to sustain Notch signaling in breast and glioma CSCs [239]. Similarly, proteasome subunits are themselves regulated by stem cell factors such as Musahi-1, which downregulates the 26 S proteasome [239], implying two-way crosstalk between stemness program and the proteasome function. Recent evidence indicates that the ulfolded protein response (UPR), a key component of ER proteostasis, not only supports bulk tumor cell survival but also contributes to CSC maintenance. The ATPase p97/VCP, which participates in ER-associated degradation and other proteostatic processes, is consistently expressed at higher levels in CD44⁺/CD24⁻ or ALDH**⁺** CSC populations than in their non-CSC counterparts, and its expression correlates with the stemness factor SOX2. Pharmacological inhibition of p97 disrupts UPR signaling and diminishes CSC self-renewal capacity [240]. Conversely, excessive activation of UPR can have the opposite effect: ER-stress-induced UPR promotes differentiation of colon CSCs and increases their sensitivity to chemotherapy both in vitro and in vivo [241]. Thus, a finely tuned, adaptive level of UPR appears to be required for CSC persistence. UPR signaling also maintains redox balance by regulating antioxidant gene expression and limiting ROS accumulation [242]. Chemotherapy-induced oxidative stress can generate senescent tumor cells with renewed stem-like activity, and reduced 26 S proteasome activity further stabilizes NRF2, facilitating the transition from a high-ROS quiescent to a low-ROS proliferative state [243]. Proteasomes can also maintain stem-like properties of CSCs by specifically degrading ubiquitin modified signal molecules [244]. Beyond the proteasome, aggrephagy, a selective form of autophagy, also contributes to protein quality control in stem cells [245]. Despite their low global protein synthesis rates, stem cells display high levels of ribosomal biogenesis [246–249]. This apparent paradox is is explained by post-transcriptional regulatory mechanisms, including limited protein synthesis through altered initiation that restricts which mRNAs are actively translated [250, 251]. In line with this concept, inhibition of translation elongation suppresses CSCs and reduces tumor growth [252], indicating that CSCs depend on tightly controlled translation rather than high translational output. Furthermore, proteasome activity is functionally linked to mitochondrial metabolism, and cells with low proteasome activity do not necessarily exhibit reduced protein synthesis, suggesting an imbalance between protein production and proteasomal degradation in these cells [253].

Interestingly, reports on proteasome activity in CSCs are not entirely consistent. Studies on CD133^+^ glioblastoma stem cells have reported elevated protein synthesis rates, potentially driven by genomic alterations or specific metabolic pathways. For example, loss of PTEN leads to constitutive activation of the PI3K/AKT/mTOR pathway, which simultaneously enhances protein synthesis and suppresses autophagy. The resulting imbalance creates a reliance on proteasomal degradation and heightened sensitivity to proteasome inhibition [254]. Consistently, threonine-dependent tRNA modification enhances global translation and selective proteome remodeling in CD133^+^ glioblastoma stem cells, linking amino acid availability to tumor growth [108]. CD133^+^ glioblastoma stem cells are markedly more sensitive to proteasome inhibitors than their differentiated counterparts due to elevated ubiquitination and ER stress–mediated apoptotic signaling. This vulnerability can be further enhanced by disrupting UPR-associated survival pathways [255]. Although CD133 is widely used to enrich glioblastoma stem cells, its reliability as a universal marker of stemness has been questioned [256]. Insights from hematological malignancies indicate that differential activation of compensatory proteostasis pathways, such as HSF1 signaling and autophagy, can determine sensitivity to proteasome inhibition (effective in multiple myeloma, but not effective in acute myeloid leukemia) [257]. Inter-tumor differences in proteasome regulation among CSCs may reflect variability in translational activity, autophagy, redox balance, and oncogenic signaling context. Highly proliferative, mTOR-activated CSCs with elevated protein synthesis may display increased dependence on proteostasis pathways, whereas more quiescent or autophagy-competent states may display reduced reliance on proteasomal degradation. These differences likely arise from stemness plasticity rather than a uniform proteasomal phenotype. Overall, CSCs appear to maintain stemness through a distinct proteostatic balance involving altered translation control, adaptive UPR signaling, and relatively low proteasome activity compared with bulk tumor cells.

## Contextual factors shaping CSC metabolism

**Fig. 4:**
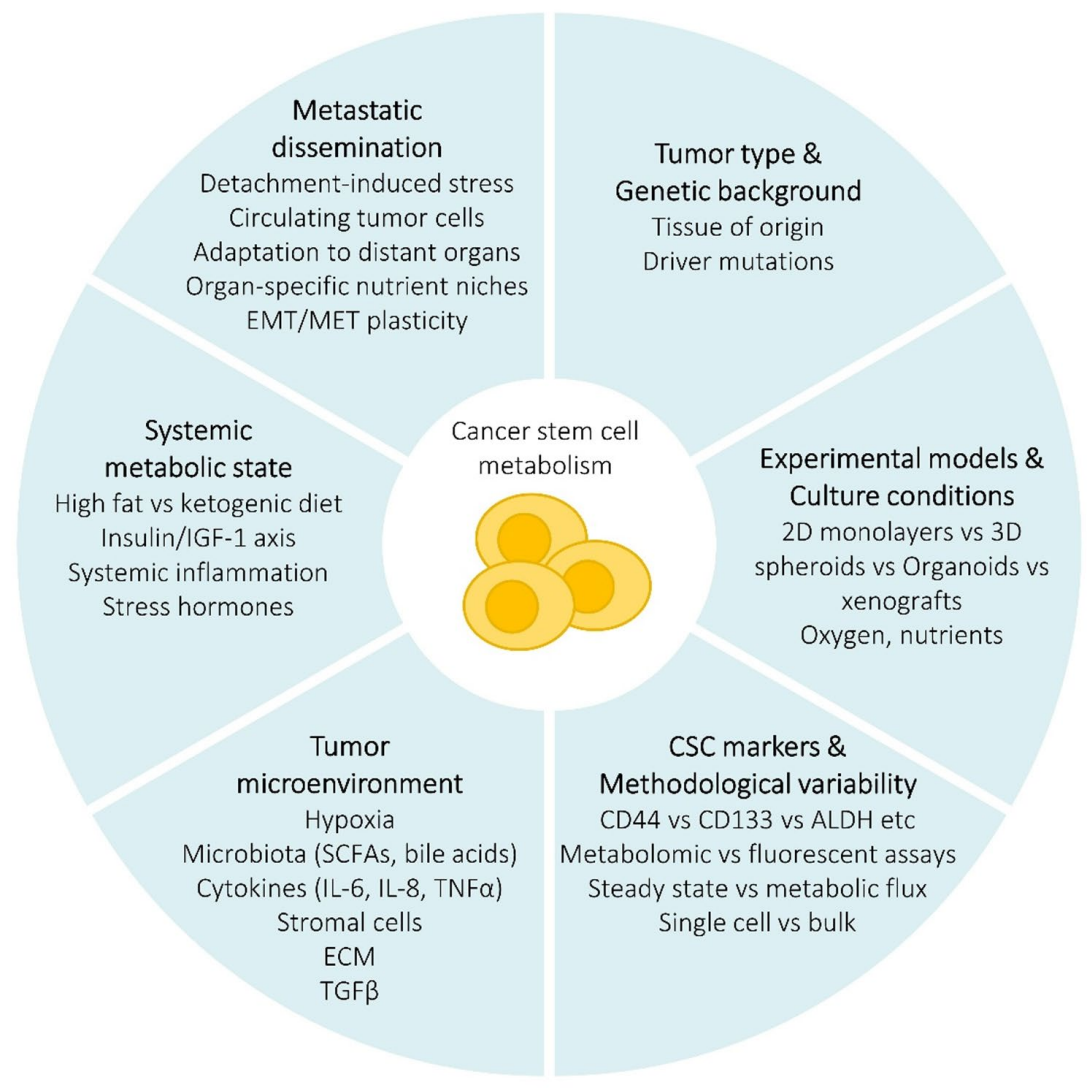
Contextual factors shaping CSC metabolism. The diagram integrates major contextual determinants that influence CSC metabolic phenotypes. Differences in tumor origin, genetic programs, culture models, and analytical methods, together with microenvironmental and systemic cues, collectively shape the metabolic behavior of CSCs. Metastatic dissemination introduces additional metabolic pressures. These interacting factors explain much of the heterogeneity observed across CSC studies. *ECM* extracellular matrix, *EMT* epithelial–mesenchymal transition, *MET* mesenchymal–epithelial transition, *SCFAs* short chain fatty acids, *TGFβ* transforming growth factor β, *TNFα* tumor necrosis factor α

CSCs display considerable metabolic heterogeneity and plasticity, and this underlies why different studies often reach apparently conflicting conclusions about their metabolic phenotype (Fig. 4).

### Model- and method- related factors

CSC metabolic profiles differ depending on the tumor type, tissue of origin and mutational background—some CSCs rely predominantly on glycolysis, while others favour OXPHOS [48]. They can also switch between glycolysis and OXPHOS, or engage fatty-acid oxidation, in response to microenvironmental stress (hypoxia, nutrient deprivation), which means that measurements capture a snapshot of a dynamic state [258]. The experimental models and culture conditions vary widely, and whether cells are grown as adherent 2D monolayers, 3D spheres, primary patient-derived cultures, or xenografts profoundly influences nutrient, oxygen, and cell-interaction contexts, and consequently their metabolism [83, 259, 260]. Moreover, the definition of CSCs is not uniform across studies: different markers (e.g., CD44, CD133, ALDH1A1) or functional assays may select different sub-populations with distinct metabolic states [15, 261]. Technical and methodological differences (e.g., how metabolism is measured, whether via Seahorse assays, metabolomics, tracer studies, or flow cytometry-based probes) and differences in data normalisation can affect interpretation of whether one pathway is “higher” or “lower” [34, 262]. Discrepancies across studies investigating CSC metabolism often arise not from methodological error but from genuine contextual diversity in how CSCs adapt to their environment. In this dynamic framework, the metabolic heterogeneity observed among CSC populations likely reflects the reversible and plastic nature of stemness, rather than the existence of a metabolically uniform subpopulation [263].

### Tumor microenvironment

CSCs do not occur in the middle of nowhere, instead, they interact dynamically with stromal cells, immune components, extracellular matrix (ECM) elements, and metabolic gradients that collectively define the niche. The tumor microenvironment (TME) plays a central role in shaping the CSC phenotype, making it context-dependent rather than fixed [264]. Stromal components of the TME, such as cancer-associated fibroblasts (CAFs) and endothelial cells secrete pro-stemness mediators such as TGF-β, hepatocyte growth factor, and soluble JAGGED-1, which induce EMT and activate Notch signaling, supporting CSC survival, invasion, and retention in specialized niches [265]. CAFs further promote CSCs via paracrine IL-6/STAT3 signaling and by releasing exosomes enriched in pro-stemness microRNAs, linking stromal signaling to CSC maintenance and therapy resistance [266, 267]. In parallel, tumor-associated macrophages (TAMs) secrete IL-6 to activate STAT3 and thereby increase the fraction of stem-like cells. Pharmacologic inhibition of IL-6/STAT3 reduces stemness and treatment resistance [268]. Pro-inflammatory cytokines, including TNF-α, IL-6, and IL-8, released by CAFs and TAMs, activate NF-κB, JAK/STAT, and COX-2 pathways, promoting CSC survival and driving the conversion of non-CSCs into CSC-like cells [269]. Beyond signaling, CAFs provide metabolic protection by producing glutathione and ROS-scavenging molecules, shielding CSCs from therapy-induced oxidative stress [270]. Reciprocal interaction between CSCs and stromal cells is mediated by hypoxia, a hallmark of rapidly growing tumors. Hypoxia stabilizes transcription factors HIF-1α and HIF-2α and rewires metabolism to favor CSC maintenance and therapy resistance by promoting self-renewal, EMT, DNA repair and stemness-associated pathways such as Notch, Hedgehog, and Wnt/β-catenin [271]. HIF signaling promotes CAF differentiation, and CAF-derived IL-6/STAT3 and Notch activation then sustain CSC survival and therapy resistance, establishing a self-reinforcing niche [272–274]. Hypoxia reprograms CAFs to increase collagen production and remodel the ECM, reinforcing a pro-tumorigenic niche. Resulting matrix stiffening enhances integrin signaling and activates YAP/TAZ–dependent mechanotransduction, which promotes CSC traits and invasion [275, 276]. In parallel, angiocrine signals from endothelial cells, particularly DLL4-Notch, support stem-like phenotypes and therapy resistance, defining a vascular niche for CSCs [265, 277]. Collectively, hypoxic, inflammatory, mechanical, angiocrine, and metabolic cues from TME converge to drive the expansion of CSC populations and undermine treatment efficacy.

### Microbial metabolites

Recent evidence indicates that host-associated microbes and their metabolites can directly modulate CSC phenotypes. Microbial metabolites such as short-chain fatty acids and secondary bile acids exert signalling and epigenetic effects on CSCs, for example by engaging the aryl hydrocarbon receptor (AhR) or by modifying histone acetylation, thereby altering self-renewal, differentiation and therapy sensitivity. In colorectal cancer, microbiome-derived formate was shown to activate AhR signalling and increase cancer stemness and metastatic behaviour, while other microbe-derived metabolites (butyrate, deoxycholate) have context-dependent effects on differentiation and stem cell programmes [278–280]. Mechanistically, microbes influence CSCs through at least three non-exclusive routes: (i) direct metabolite signalling that provides alternative carbon sources and epigenetic cofactors (e.g., formate, short chain fatty acids, bile acids), (ii) microbe-host receptor signalling (Toll-like receptors, AhR) and activation of canonical stemness pathways (Wnt, Notch, STAT3), and (iii) genotoxic or inflammatory actions that reshape the tumor evolutionary landscape (e.g., colibactin-associated mutational signatures). These axes are best evidenced in gastrointestinal malignancies but emerging data show intratumoral bacteria and microbiome-tumor crosstalk in breast, pancreatic and lung cancers, with translational implications for biomarker discovery and therapeutic modulation of CSC pools [281, 282]. Although evidence remains preliminary, microbial metabolites such as short-chain fatty acids, secondary bile acids, and tryptophan derivatives are emerging as host-level modulators capable of reshaping CSC metabolism and phenotypic plasticity.

### Systemic physiological and dietary regulators

Systemic dietary and neuroendocrine factors provide an additional regulatory layer shaping CSC metabolism and plasticity. High-fat diet enhances intestinal stemness and tumorigenicity through a PPARδ- and PPARα- driven fatty acid oxidation and inhibition of CPT1 suppresses these effects [283]. Ketogenic diet attenuates proliferation and stemness of glioma stem-like cells by altering metabolism fluxes and increasing ROS levels [191]. Insulin/IGF signaling regulates breast CSCs self-renewal via IRS2- and PI3K-dependent stabilization of MYC [284]. Stress hormones further modulate CSC states—norepinephrine induces β2-adrenergic IL-6 production in breast cancer cells, which subsequently promotes cancer cell migration [285]; chronic epinephrine increases LDH activity and expands breast CSC populations [286]; and sustained norepinephrine exposure enhances stem-like phenotypes in oral cancer via β2-adrenergic receptors [287]. Alongside these stress-mediated effects, sex-specific hormonal and metabolic backgrounds shape CSC behaviour, as androgens and estrogens differentially modulate oxidative metabolism, inflammatory signaling and epigenetic programs. Male cells generally rely more on glucose and amino acids, whereas female cells preferentially utilize lipids and show enhanced fatty-acid oxidation. Female mitochondria exhibit higher respiratory activity but accumulate less ROS due to stronger antioxidant defenses, whereas male mitochondria generate more ROS and display lower oxidative resilience [288]. Additional systemic endocrine regulators also influence CSC states—glucocorticoids promote quiescence, chemoresistance and stem-like transcriptional programs in breast and brain cancers [289]. Thyroid hormone promotes cell self-renewal in hepatocellular carcinoma cells [290], and thyroid hormone receptor β modulates CSCs activity in anaplastic thyroid cancer [291]. Finally, circadian rhythm disruption alters metabolic fluxes and epigenetic clocks in stem and progenitor populations [292], while aging remodels mitochondrial function, metabolite availibility and epigenetic stability [293]. Collectively, these systemic modifiers suggest that CSC metabolism cannot be fully understood in isolation from systemic physiological context.

### Metastatic dissemination and organ-specific metabolic adaptation

Metastatic dissemination represents one of the most stringent metabolic selection pressures encountered by CSCs, requiring rapid and reversible metabolic reprogramming across the metastatic cascade (invasion—circulation—colonization) and the adaptation to specific organs (lung, brain, liver, bone, etc.). EMT enables metastatic dissemination by enhancing cellular motility, invasiveness, and resistance to detachment-induced stress, whereas subsequent MET supports efficient colonization at distant sites [43]. To form metastases, cancer cells must detach from the ECM and enter the blood or lymphatic stream, where survival under anchorage-independent growth is required. However, only a very small proportion of circulating cancer cells (less than 0.1%) are able to survive and form metastases [294]. Detachment from the ECM induces oxidative stress and represents a major challenge for disseminating cells [295], which can partially overcome this stress by forming clusters or aggregates that enhance survival during circulation. Cell clustering induces hypoxia and activates mitophagy to remove damaged mitochondria, while reductive carboxylation supports glycolytic metabolism. These responses reduce mitochondrial ROS production and promote metastasis [296]. Nevertheless, controlled increases in ROS can act as signaling molecules that promote EMT and metastatic dissemination. Accordingly, CSC populations characterized by elevated mitochondrial ROS have been associated with enhanced metastatic capacity [177]. Several metabolic pathways contribute to metastatic competence, including amino acid metabolism, pyruvate, lactate and acetate utilization, the pentose phosphate pathway, and glutathione synthesis [297, 298]. For example, metastatic breast cancer cells utilize lactate as a mitochondrial fuel following detachment, sustaining oxidative phosphorylation and stemness through α-ketoglutarate-mediated epigenetic regulation of SOX2 [299]. Similarly, fatty acid synthesis mediated by FASN has been shown to support survival under metabolic and oxidative stress associated with ECM detachment [300]. Metastatic cancer cells may also rely on monocarboxylate transporter-1 (MCT1)-mediated lactate uptake to support energy production and oxidative stress tolerance, and inhibition of MCT1 reduces metastatic potential [301]. Successful colonization of distant organs requires a metabolic shift from stress adaptation to sustained proliferation and biomass production—increased mitochondrial fitness, enhanced oxidative phosphorylation, and reactivation of anabolic pathways, including nucleotide, lipid, and amino acid biosynthesis. Metastasis-initiating breast cancer cells rely on oxidative metabolism to sustain metastatic potential. In these cells, mitochondria-derived citrate supports acetyl-CoA production and H3K27 acetylation, thereby promoting EMT-related gene expression and metastatic colonization [302]. Organ-specific microenvironments further shape metastatic metabolism. For example, brain metastases are able to utilize glutamine, BCAAs, and acetate as carbon sources to fuel the TCA cycle, whereas lung metastases can exploit pyruvate to drive collagen-based ECM remodeling in the metastatic niche. Liver metastases often exhibit a more glycolytic phenotype, while bone metastases rewire their metabolism through osteopontin-mediated programs that regulate glucose and glycerol utilization and support serine production. These organotropic adaptations indicate that metastatic success depends not only on intrinsic plasticity but also on the ability to align cellular metabolism with tissue-specific metabolic environments [303, 304]. In addition, disseminated tumor cells may enter a dormant state characterized by reversible G_0_ cell cycle arrest and distinct metabolic adaptations that enable long-term survival and eventual metastatic relapse. Dormant disseminated tumor cells often rely on mitochondrial metabolism, including oxidative phosphorylation and fatty acid oxidation, together with enhanced antioxidant pathways that maintain redox balance and support persistence within organ-specific niches [305, 306]. These observations indicate that CSC-associated metabolism is highly dynamic during metastatic progression and reported metabolic phenotypes may therefore reflect transient metabolic states rather than the existence of a metabolically uniform CSC population.

## Translational outlook: metabolic targeting of CSCs

Most metabolic inhibitors evaluated to date have been tested primarily in bulk tumor cell populations, and only a subset of studies has directly examined their effects on CSCs, highlighting the need for functional CSC readouts in translational research. As a result, much of the current clinical evidence reflects tumor-wide metabolic dependencies rather than CSC-specific vulnerabilities [48, 307, 308]. Clinical translation faces two central challenges: (1) metabolic plasticity that enables CSCs to compensate for blocked pathways, and (2) toxicity/selectivity constraints because many metabolic enzymes are shared with normal tissues. Resistance to metabolic therapy commonly arises from metabolic plasticity: CSCs switch substrates (e.g., from glutamine to fatty acids or from glycolysis to OXPHOS) or increase uptake of stromal-derived metabolites to bypass the inhibited node. Additional mechanisms include redundancy among enzyme isoforms/transporters and microenvironmental rescue (stromal cells or macrophages supplying lactate, alanine, fatty acids). Understanding these escape routes is critical for designing combinations that prevent metabolic compensation [307]. The most extensively explored approach involves inhibition of mitochondrial OXPHOS, as CSCs in many tumor types rely on mitochondrial respiration for ATP generation and survival. Agents such as Metformin or Phenformin reduce the TCA and selected glycolytic intermediates and selectively eliminate OXPHOS-dependent CSCs [309, 310]. A second major strategy targets fatty acid metabolism, which sustains CSC energy demands and membrane synthesis. Inhibitor of CPT1 block fatty acid oxidation and impair CSCs self-renewal [311, 312], while inhibitors of FASN or SCD1 reduce stemness markers, sphere formation, and metastatic potential [131, 313, 314]. Targeting lipid uptake and transport, particularly through CD36 [315] and related enzyme ACC [316], has also shown promise in depleting lipid-addicted CSC population. The ALDH/Nrf2-redox axis represents another critical vulnerability. ALDH enzymes detoxify reactive aldehydes and maintain low ROS levels; pharmacological inhibition or Nrf2 knockdown diminishes the ALDH⁺ CSC fraction, sensitizing tumors to chemo- and radiotherapy [190].

Across tumor types, pairing chemotherapy with a metabolic inhibitor (Complex I, FAO/CPT1, SCD1, ALDH) may further enhance CSC depletion by restoring therapy sensitivity. Combining a fatty acid oxidation inhibitor and Gemcitabine treatment enhanced drug efficacy in vitro and in vivo, effectively diminishing the CSC content and functionality [317]. The combination of Metformin and a chemotherapeutic agent Doxorubicin eliminated both CSCs and non-stem cancer cells, reduced tumor mass and prevented relapse much more effectively than either drug alone in a xenograft mouse model [318]. Analysis of leukemia stem cells from patients undergoing treatment with Bcl-2 inhibitor Venetoclax and DNA methyltransferase inhibitor Azacitidine showed disruption of the TCA cycle manifested by decreased α-ketoglutarate and increased succinate levels which suppress OXPHOS and selectively targets leukemia stem cells [319]. SCD1 inhibitors significantly enhance sensitivity of bladder CSCs to Cisplatin treatment [320]. Detailed overview of current therapeutic trials, inhibitors, combination therapies, limitations, resistance mechanisms and clinical relevance has been published recently [49]. These studies highlight that disrupting mitochondrial energy production, lipid synthesis and oxidation, and redox homeostasis can effectively compromise CSC maintenance and represents a promising translational avenue for metabolic targeting in cancer therapy.

## Conclusion & future perspectives

CSCs do not rely on a single metabolic identity but instead maintain a flexible network of mitochondrial, glycolytic, lipid, redox, and proteostatic circuits that enable survival and adaptation across diverse microenvironmental conditions. Their metabolic heterogeneity, together with TME-derived cues and therapy-induced reprogramming, creates a shifting landscape that challenges conventional targeting strategies. Although major advances have been made in understanding CSC metabolism, there are several key gaps and unresolved questions in the CSC field that limit the progress:

- Marker heterogeneity—no marker is universal; marker-based, functional, transcriptomic, or metabolic definitions often diverge. CD44, CD133, ALDH1A1, EpCAM etc. select different subpopulations even within a single tumor.
- Poorly mapped boundaries of CSC plasticity and state transitions in vivo—a large part of CSC properties is a state, not a stable population, it is still unclear which signals trigger CSC to non-CSC transitions in vivo and how stable these states are.
- Unclear in vivo CSC frequencies—CSC frequency varies dramatically depending on the xenograft model, host immune status etc.
- Metabolic contradictions—no clear explanation for OXPHOS-high vs. glycolytic, glutamine-dependent vs. glutamine-independent, FAO-driven vs. lipogenic phenotype.
- Contextual dependency—tumor type, mutational background, oxygenation, ECM, cytokines, microbiota, dietary factors, immune cells—all of these can modify CSC metabolism, often in opposite directions and only a few studies test multiple conditions in parallel.
- Need for spatial, single-cell, and metabolic multi-omic data—many key conclusions are based on 2D cell lines and immunosuppressed mice. These models do not include immune pressure, stromal interactions, and metabolic limits.
- Limited clinical evidence—most metabolism-targeting therapies for CSCs have failed or never reached the clinic, we do not know which metabolic phenotypes patients actually have, because human biopsy metabolomics is rare.

Addressing these gaps will be essential for translating metabolic insights into clinically meaningful CSC-directed therapies.

## Data Availability

No datasets were generated or analysed during the current study.

## Abbreviations

ACC: Acetyl-CoA carboxylase
ACLY: ATP citrate lyase
ACS: Acyl-CoA synthetase
AhR: Aryl hydrocarbon receptor
ALDH: Aldehyde dehydrogenase
ASCT2: Alanine/Serine/Cysteine Transporter 2
BCAA: Branched-chain amino acid
BCAT1/2: Branched-chain aminotransferases
CAF: Cancer associated fibroblast
CSC: Cancer stem cell
CPT1: Carnitine palmitoyltransferase 1
ECM: Extracellular matrix
EMT: Epithelial–mesenchymal transition
FASN: Fatty acid synthase
FAO: Fatty acid oxidation
GLUT: Glucose transporter
HAT: Histone acetyltransferase
HDAC: Histone deacetylase
HSP: Heat shock protein
IDH: Isocitrate dehydrogenase
LDH: Lactate dehydrogenase
m^6^A: *N*^6^-methyladenosine
MET: Mesenchymal–epithelial transition
MMP: Mitochondrial membrane potential
NFκB: Nuclear factor κB
PPAR: Peroxisome proliferator-activated receptor
ROS: Reactive oxygen species
SCD1: Stearoyl-CoA desaturase-1
SDH: Succinate dehydrogenase
SREBP: Sterol regulatory element-binding protein
OXPHOS: Oxidative phosphorylation
SDH: Succinate dehydrogenase
TAMs: Tumor-associated macrophages
TCA: Tricarboxylic acid cycle
TME: Tumor microenvironment
UPR: Unfolded protein response
